# Pyogenic Spondylitis Four Years After an Injection Into the Paraspinal Muscles

**DOI:** 10.7759/cureus.64810

**Published:** 2024-07-18

**Authors:** Yamamoto Shinta, Masatochi Hori, Hiroshi Okada

**Affiliations:** 1 Internal Medicine, Matsushita Memorial Hospital, Osaka, JPN; 2 Emergency Medicine, Matsushita Memorial Hospital, Osaka, JPN

**Keywords:** mssa bacteremia, lower back pain, unexplained fever, delayed abscess, pyogenic spondylitis

## Abstract

In the context of local injection techniques, there are infectious complications such as epidural abscesses and pyogenic spondylitis. An 83-year-old female who received an injection into the paraspinal muscles for lower back pain four years ago developed a paraspinal muscle abscess accompanied bymethicillin-susceptible *Staphylococcus aureus *bacteremia and multiple sites of pyogenic spondylitis. Non-surgical treatment was followed with 73 days of antibiotic therapy by cefazoline. We noted an improvement in inflammatory response in blood tests and findings of inflammation on imaging tests. Instances of delayed-onset infectious complications several years after simple local injection techniques are rare.

## Introduction

Pyogenic spondylitis is a relatively rare condition with an incidence rate of approximately 5.3 to 7.4 patients per 100,000 person-years, as indicated by a retrospective cohort study using the Diagnosis Procedure Combination data in Japan [1]. Treatment generally requires rest and adequate doses of sensitive antibiotics. Its etiology includes hematogenous spread from distant infectious foci, direct dissemination to the spine through trauma, medical procedures, or surgery, as well as secondary propagation from adjacent soft tissue infections. Risk factors encompass a history of spinal surgery (including degenerative spinal disorders) and underlying conditions that compromise the immune system, such as diabetes and corticosteroid usage. Additionally, the use of injectable medications has been identified as a potential factor [2,3]. While local injection techniques such as trigger point injections or nerve root blocks have been reported to cause intramuscular abscesses or epidural abscesses, infectious complications from such procedures usually occur within days to up to six months [4,5].

In this case, a patient with no underlying condition developed spondylitis. Moreover, the onset of spondylitis was considered to be attributed to a lumbar injection performed four years prior.

## Case presentation

The patient was an 83-year-old female with spinal canal stenosis. She had no history of atopic dermatitis, no recent history of tooth extraction, and no significant family or social history. She was not on any medication or supplemental agents. Four years ago, she had visited a local doctor for treatment of lower back pain and had received an injection in the lumbar region. Thereafter, there was no history of trauma. She arrived at our hospital via ambulance due to loss of appetite and weakness in the lower limbs, which had started three days before.

The patient had a height of 149 cm and a weight of 49.4 kg. She was alert and oriented and had no abnormal findings regarding breath sounds or heart sounds and no sensory deficits or paralysis in the limbs. Laboratory tests showed elevated inflammatory markers with a white blood cell count of 13,800/μL and C-reactive protein of 34.55 mg/dL. Procalcitonin was slightly elevated at 2.51 ng/mL. The test was positive for nitrite and white blood cells in the urine dipstick test (Table 1). Gram staining of urine showed the presence of Gram-positive cocci.

**Table 1:** Laboratory results of the patient.

Blood test		Reference range	Urinalysis		Reference range
White blood cell	13,800/μL	3,300–8,600/μL	Specific gravity	1.023	1.010–1.030
Neutrophils	88.90%		pH	5.5	4.8–7.5
Lymphocytes	5.40%		Glucose	−	
Eosinophils	0%		Blood	3+	
Monocytes	5.70%		Ketones	2+	
Red blood cell	484 × 10^4^/μL	386 × 10^4^–492 × 10^4^/μL	Protein	2+	
Hemoglobin	15.0 g/dL	11.6–14.8 g/dL	Urobilinogen	+-	
Platelets	13.3 × 10^3^/μL	158 × 10^3^–348 × 10^3^/μL	Bilirubin	-	
Sodium	132 mEq/L	137–147 mEq/L	White blood cells	30–49/HPF	
Potassium	3.5 mEq/L	3.5–5.0 mEq/L	Red blood cells	>100/HPF	
Chloride	95 mEq/L	98–108 mEq/L	Nitrites	2+	
Calcium	9.2 mg/dL	8.4–10.4 mg/dL	Bacteria	2＋	
Aspartate aminotransferase	85 U/L	8–38 U/L	Yeast	–	
Alanine aminotransferase	59 U/L	4–43 U/L			
γ-glutamyl transpeptidase	77 U/L	<48 U/L			
Alkaline phosphatase	216 U/L	38–113U/L			
Total bilirubin	1.7 mg/dL	0.2–1.2 mg/dL			
Lactate dehydrogenase	336 U/L	124–222 U/L			
Creatine kinase	419 U/L	30–172 U/L			
Total protein	7.4 g/dL	6.5–8.3 g/dL			
Albumin	3.2 g/dL	3.8–5.3 g/dL			
Urea nitrogen	54 mg/dL	8–20 mg/dL			
Creatinine	1.08 mg/dL	0.47–0.79 mg/dL			
C-reactive protein	34.55 mg/dL	<0.30 mg/dL			
Serum glucose	139 mg/dL	60–109 mg/dL			
Procalcitonin	2.51 ng/mL	<0.05 ng/mL			
Prothrombin time	92%	70–120%			
Activated partial thromboplastin time	28.9 seconds	24.0–34.0 seconds			
D-dimer	32.4 μg/mL	<1.0 μg/mL			
Fibrinogen	842 mg/dL	200–400 mg/dL			

**Figure 1:**
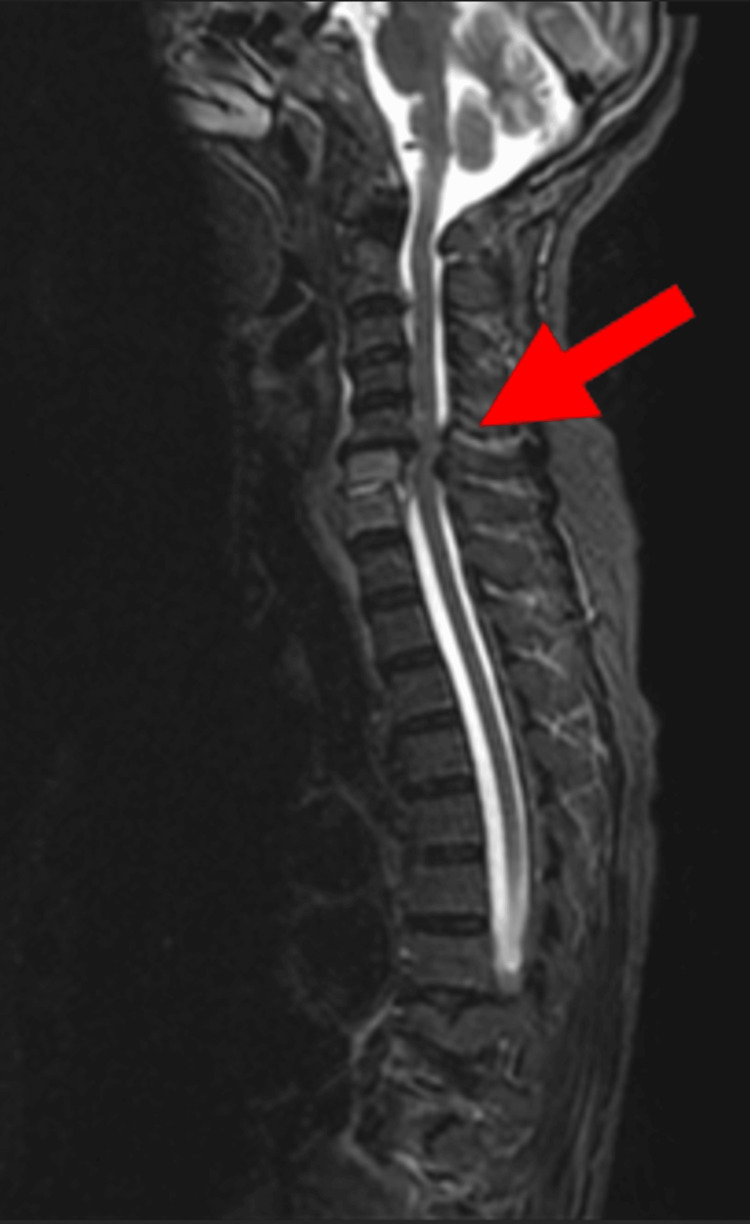
MRI findings of the entire spine. Short inversion time inversion recovery revealing a new high intensity at the L1 and L2 vertebral bodies (arrow).

**Figure 2:**
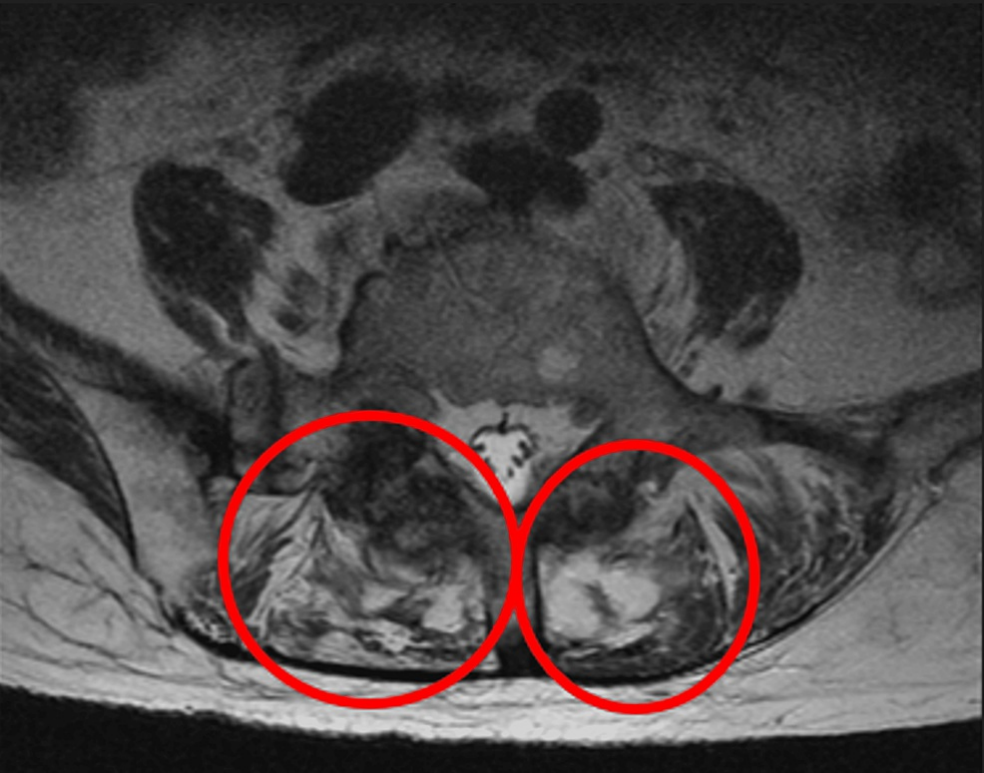
T2 revealing fluid collection in the paraspinal muscle adjacent to the upper L5 vertebral body (circle).

The possibility of sepsis was suspected due to elevated hepatic enzymes and worsening renal function. An abdominal and chest CT scan was performed, and no abnormalities that could account for the elevated inflammatory markers were observed. Antibiotic therapy was initiated with sulbactam sodium/ampicillin sodium 9 g/day and vancomycin 1 g/day for suspected urinary tract infection. Owing tomethicillin-susceptible *Staphylococcus aureus* being detected from blood and urine cultures obtained at admission, we de-escalated antibiotics to cefazolin sodium 3 g/day from the second day of hospitalization. We initially suspected infective endocarditis and performed transesophageal echocardiography, but there were no obvious abnormal findings. On examination after admission, the patient complained of back pain. We performed an MRI of the entire spine which revealed high intensity in the C6/7 intervertebral disc and fluid collection in the paraspinal muscle adjacent to the upper L5 vertebral body (Figures 1, 2).

**Figure 3:**
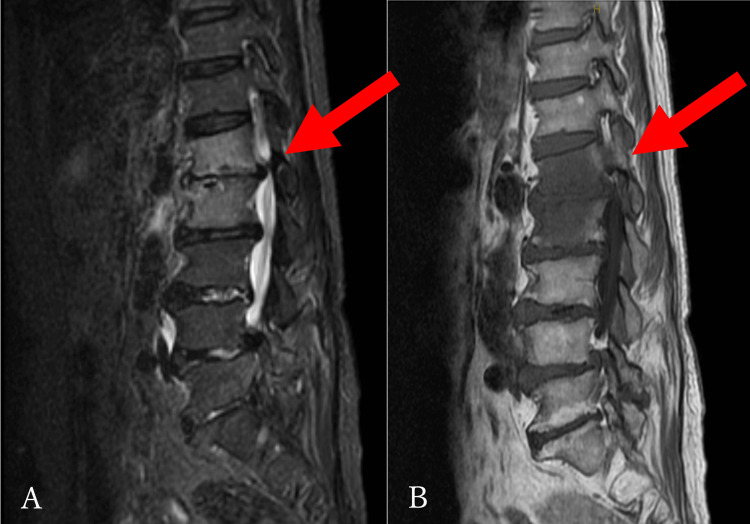
MRI findings of the entire spine. A, B: Short inversion time inversion recovery revealing a new high intensity at the L1 and L2 vertebral bodies, with T1 revealing a low intensity (arrow).

Based on these findings, a diagnosis of pyogenic spondylitis at C6 and C7 and paraspinal muscle abscess was made. On the 16th day of hospitalization, a follow-up MRI showed improvement in the abscess; however, a new region of pyogenic spondylitis at the L1 and L2 vertebral bodies was identified (Figure 3).

Consultations were held with the orthopedic and neurosurgery departments regarding abscess drainage and spinal fixation surgery. Due to the small size of the abscess and the absence of neurological abnormalities, the decision was made to continue conservative management with intravenous antibiotic administration and the application of a cervical/lumbar brace [6]. Blood cultures were retested every three to seven days, and on the 61st day of hospitalization, they turned negative, leading to a switch to oral antibiotics by cephalexin for 35 days. Subsequently, the patient’s condition progressed without fever or increased inflammatory response. Reassessment through MRI showed the resolution of the paraspinal muscle abscess and improvement in vertebral inflammation. Rehabilitation therapy was continued, and the patient was discharged on the 113th day of hospitalization.

## Discussion

Pyogenic spondylitis often results from hematogenous infections, which can be attributed to the absence of valve structures in Batson’s venous plexus around the spine, allowing easy dissemination of bacteria from abscesses [7]. Moreover, there have been several reports of abscess formation following trigger point injections and nerve root blocks [8].

We diagnosed pyogenic spondylitis that could be caused by an injection performed four years prior. Regarding the timing of such infectious complications, in a report of 18 cases of abscess formation after nerve root injections in non-immunocompromised patients, the time between injection and diagnosis varied from 3 to 120 days, with most cases being diagnosed around 30 days [4]. In addition, surgical site infections, which are common in terms of infection following external invasion, occur within 30 days after surgery in most cases [9]. However, it is worth noting that a case has been reported of a man who developed delayed pyogenic spondylitis and psoas muscle abscess five years after undergoing percutaneous lumbar correction surgery [10]. This suggests the possibility of delayed abscess formation even after injection procedures. We consider the possibility that a microabscess may have developed from a small puncture wound.

Based on the above, our case was diagnosed as multiple sites of pyogenic spondylitis due to hematogenous dissemination from paraspinal muscle abscess as a rare delayed complication of injection therapy for lower back pain. There is no literature on abscess formation several years after injection procedures. Additionally, it is intriguing that our patient had no underlying conditions besides spinal canal stenosis and no history indicating immunodeficiency, yet developed delayed infectious complications.

## Conclusions

This case highlights two points, namely, the possibility of developing delayed abscess and pyogenic spondylitis even four years after a paraspinal muscle injection, and the fact that such delayed infectious complications can occur even in patients without immunodeficiency. We suggest inquiring about medical history dating back quite a while to investigate the cause of unexplained fever or search for the source of bacteremia.
